# High content of long-chain n-3 polyunsaturated fatty acids in red blood cells of Kenyan Maasai despite low dietary intake

**DOI:** 10.1186/1476-511X-10-141

**Published:** 2011-08-19

**Authors:** Nadja Knoll, Katrin Kuhnt, Florence M Kyallo, Beatrice N Kiage-Mokua, Gerhard Jahreis

**Affiliations:** 1 Department of Nutritional Physiology, Institute of Nutrition, Friedrich-Schiller-University, Dornburger Str. 24, Jena, Germany; 2 Department of Food Science and Technology, Jomo Kenyatta University of Agriculture and Technology, Nairobi, Kenya

**Keywords:** Maasai diet, Maize meal, Milk intake, red blood cell fatty acids

## Abstract

**Background:**

Increasing land restrictions and a reduced livestock-to-human ratio during the 20th century led the Maasai to lead a more sedentary, market-orientated lifestyle. Although plant-derived food nowadays contributes substantially to their diet, dairy products being high in saturated fatty acids (SFA) and low in polyunsaturated fatty acids (PUFA) still are an important energy source. Since reliable data regarding the Maasai diet date back to the 1980s, the study objective was to document current diet practices in a Kenyan Maasai community and to investigate the fatty acid distribution in diet and red blood cells.

**Methods:**

A cross-sectional study was conducted among 26 Maasai (20 women, 6 men) from Loodokilani, Kajiado District, Kenya. Food intake was described by the subjects via 24-h recall, and both food and blood samples were analysed.

**Results:**

Two main foods - milk and *ugali *- constituted the Maasai diet in this region. A total of 0.9 L of milk and 0.6 kg of *ugali *were consumed per person and day to yield an energy intake of 7.6 MJ/d per person. A major proportion of ingested food contributing 58.3% to the total dietary energy (en%) was plant-derived, followed by dairy products representing 41.1 en%. Fat consumed (30.5 en%) was high in SFA (63.8%) and low in PUFA (9.2%). Long-chain n-3 PUFA (EPA, DPA and DHA) made up only 0.15% of the ingested fatty acids, but 5.9% of red blood cell fatty acids.

**Conclusion:**

The study indicates the Maasai diet is rich in SFA and low in PUFA. Nevertheless, red blood cells are composed of comparable proportions of long-chain n-3 PUFA to populations consuming higher amounts of this fatty acid group.

## Background

The agro-pastoral tribes of the Maasai inhabit territories in northern Tanzania and southern Kenya. During the 1960s and 1970s, various reports described the Maasai as subsisting on a diet of excessive amounts of milk and meat and thereby ingesting large quantities of fat and cholesterol [1,2]. Even today, this image of the Maasai diet, including the consumption of cattle blood, prevails (e.g., [3,4]).

During the 20^th ^century, increasing land restrictions and a reduced livestock-to-human ratio led the Maasai to lead a more sedentary, market-orientated lifestyle and for the past decades consumption of food from plant sources has gradually increased contributing substantially to their diet [5,6].

Despite this increasing ingestion of plant-derived food, dairy fat, high in saturated fatty acids (SFA) and low in polyunsaturated fatty acids (PUFA), is still the main dietary fat source [7]. For traditional and geographical reasons, sea fish containing high levels of long-chain n-3 PUFA (n-3 LC-PUFA) is not part of the Maasai diet [8,9]. These fatty acids, however, are important in humans for the development of the brain and retina and also health in general [10].

Since the only reliable and complete data regarding the Maasai diet date back to the 1980s [7,8], we aimed to document current diet practices of the Loodokilani Maasai of the Kajiado District in Kenya. In addition, we investigated the extent to which the allegedly high dietary proportion of SFA and low proportion of PUFA was reflected in the red blood cells of the Maasai.

## Methods

### Fieldwork

This cross-sectional study was undertaken at the end of the short dry season in April 2007. Maasai settlements in the Loodokilani area, situated 15 km south-west of Kajiado Town (Kajiado District), were chosen as study sites. A total of 20 healthy women (31.5 ± 9.2 years) and six healthy men (36.8 ± 18.2 years) from nine different settlements were recruited for the study. Subjects were selected according to the following criteria: voluntary participation; ages between 18-54 years; native Maasai with a traditional life-style and resident of Loodokilani for a period of more than six months; and neither pregnant nor lactating women. Volunteers meeting these criteria were informed of the purpose of the investigation in their native language, *o Maa*. All Maasai recruited for the study provided informed consent by means of a left thumb fingerprint on the consent form. The study was approved by the Kenya Medical Research Institute's (KEMRI) National Ethical Review Committee during the 142^nd ^meeting in 2007.

Each subject was visited on two consecutive days by the researchers. On the day 1, the dietary intake was recorded via 24-h recall. Consumed liquids (e.g., milk or tea) and portions of solid foods (e.g., beans) were generally indicated in mL, and were estimated by measuring the volume of the applied utensils (e.g., cups or spoons). Subjects provided information regarding the amount of ingredients used during food preparation.

On day 2, a phlebotomist from the Kajiado District Hospital took blood samples by venipuncture using 9 mL Li-heparin Monovette^® ^tubes (Sarstedt AG & Co., Nümbrecht, Germany). A total of 18 subjects from the study population provided a blood sample and were considered the sub-group of blood donors (14 females and 4 males). The remaining subjects (5 females and 3 males) did not supply a blood sample either because they were out of their homestead fulfilling their daily obligations (i.e., herding animals, fetching water or firewood) or were mistrustful regarding the purpose of the investigation. Nevertheless, the sub-group of blood donors (n = 18) can be considered representative for the total study population (n = 26) as demographical data (i.e., age, number of children and wives/co-wives), gender distribution (ca. 80% women), and food intake (each food and nutrient) did not differ significantly between blood donors and non-blood donors. Blood samples were transported in cool boxes to the Jomo Kenyatta University of Agriculture and Technology (JKUAT), Juja near Nairobi without delay and centrifuged to separate erythrocytes and plasma. Red blood cells were dispersed in NaCl solution (0.9%) and washed three times.

Food samples were collected for analyses and evaluation of the 24-h recall on both consecutive days. The Maasai provided samples of fresh (boiled) cow's milk (n = 4), fermented cow's milk (n = 8), *ghee *(i.e., home-made butter oil, n = 2), sweetened milk tea (n = 2), and suet (n = 1). In addition, *ugali *(i.e., a solid mush of maize meal; n = 3), *Kimbo*^® ^(i.e., a commercial palm oil-derived shortening used instead of *ghee *to prepare the dishes; n = 3), and raw beef (n = 1) were purchased for analyses by the researchers in local food markets. Food samples were transported to JKUAT in cool boxes. Both red blood cells and food samples were stored at -80°C and shipped to Germany within three days. Red blood cells were stored at -80°C until further analysis.

### Analyses

All food samples, except for the fats, were freeze-dried immediately on arrival in Germany. Fat, protein, and fibre content were later determined according to the methods recommended by the Association of Official Analytical Chemists [11]. Cholesterol content of food samples was ascertained by means of an enzymatic-photometric approach using a test kit (Boehringer Mannheim/R-Biopharm AG, Darmstadt, Germany). Physical energy was determined calorimetrically (Parr 1261 bomb calorimeter, Parr Instrument Co., Frankfurt/M, Germany). Total digestible carbohydrates in foods were calculated as the energy difference by subtracting the values for physical energy of fat, protein, and fibre from the calorific values. For fatty acid analyses, lipids from red blood cells and food samples were extracted by means of chloroform/methanol/water (2:1:1; v/v/v). Fatty acid methyl esters (FAME) were synthesised with sodium methylate (milk, *ghee*, and *Kimbo*^®^) and boron trifluoride in methanol (meat, *ugali*, and red blood cells). Distribution of FAME was analysed on two different gas chromatographic columns as well as using the flame ionization detector [12]. Distribution of conjugated linoleic acid (CLA) isomers in red blood cells, milk, and meat samples was determined using Ag^+^-HPLC according to Kuhnt et al. [13]. Fatty acids are expressed as % of total FAME (= g/100 g total FAME).

### Evaluation of dietary recall

Indications of liquids in mL (i.e., milk, sweetened milk tea and *porridge*) were directly converted into g, at a density of approximately 1 g/mL. Solid food mass (i.e., *ugali*, beans and vegetables) was determined by preparing the food as stated by the subjects and weighing the consumed volume. Each food/ingredient was determined separately to calculate the total intake of nutrients. The content of energy and nutrients (including the fatty acid distribution) of the collected food samples that were analysed (Table 1) were included in the evaluation of the 24-h recall. Data for foods which were not analysed (i.e., fruits, vegetables, rice, and beans) were obtained from the US Department of Agriculture (USDA) National Nutrient Database for Standard Reference [14].

**Table 1:** Composition of foods including fatty acid distribution

	Cow's milk (n = 4)		Fermented Cow's Milk (n = 8)		Ugali^1^ (n = 3)		Kimbo^®2^ (n = 3)	
	
	Mean	sd	Mean	sd	Mean	sd	Mean	sd
Energy (kJ/100 g)	**350**	7	**387**	62	**477**	17	**3930**	5
Carbohydrates^3 ^(g/100 g)	**5.4**	0.1	**5.3**	0.4	**19.9**	0.7	**0**	0
Fat (g/100 g)	**4.5**	0.2	**5.1**	1.4	**1.0**	0.0	**99.7**	0.7
Protein (g/100 g)	**3.7**	0.3	**4.3**	0.7	**2.4**	0.1	**0.3**	0.1
Fibre (g/100 g)	**--**	--	**--**	--	**2.5**	0.1	**--**	--
Cholesterol (mg/100 g)	**8.5**	1.2	**10.2**	4.3	**--**	--	**1.7**	0.1

Fatty acids (% of total FAME)								
∑C6 > C10	**6.79**	0.49	**7.81**	1.75	**0.03**	0.00	**0.03**	0.00
C12:0	**3.15**	0.09	**3.20**	0.47	**0.14**	0.01	**0.01**	0.00
C14:0	**11.67**	0.53	**11.35**	1.16	**0.05**	0.00	**0.87**	0.03
C16:0	**33.81**	1.58	**32.37**	2.37	**12.92**	0.27	**45.71**	0.45
C16:1	**1.91**	0.40	**1.74**	0.41	**0.14**	0.00	**0.14**	0.00
C18:0	**9.26**	1.07	**9.61**	1.28	**2.17**	0.05	**4.00**	0.11
C18:1*cis*9	**17.24**	1.26	**17.26**	2.03	**32.65**	0.12	**38.19**	0.51
∑C18:1*trans*	**2.30**	0.31	**2.48**	0.37	**0.04**	0.00	**0.08**	0.03
C18:1*t*11	**1.39**	0.22	**1.51**	0.29	**0.00**	0.00	**0.00**	0.00
C18:2n-6	**0.56**	0.10	**0.59**	0.15	**48.97**	0.15	**9.21**	0.17
∑CLA	**1.01**	0.14	**1.08**	0.25	**0.03**	0.00	**0.04**	0.00
c9,*t*11 CLA	**0.81**	0.11	**0.88**	0.23	**0.01**	0.00	**0.01**	0.00
C18:3n-3	**0.47**	0.11	**0.50**	0.10	**1.03**	0.01	**0.10**	0.01
C20:0	**0.34**	0.02	**0.35**	0.05	**0.46**	0.01	**0.32**	0.01
C20:4n-6	**0.05**	0.01	**0.06**	0.01	**0.00**	0.00	**0.00**	0.00
C20:5n-3	**0.06**	0.01	**0.08**	0.02	**0.01**	0.00	**0.00**	0.00
C22:0	**0.18**	0.02	**0.18**	0.02	**0.14**	0.00	**0.06**	0.00
C22:5n-3	**0.11**	0.01	**0.11**	0.02	**0.00**	0.00	**0.00**	0.00
C22:6n-3	**0.01**	0.00	**0.02**	0.01	**0.00**	0.00	**0.00**	0.00

∑SFA	**73.57**	0.90	**73.40**	2.56	**16.20**	0.23	**51.38**	0.58
∑MUFA	**23.90**	0.67	**23.89**	2.49	**33.73**	0.12	**39.26**	0.46
∑PUFA	**2.52**	0.30	**2.71**	0.37	**50.07**	0.14	**9.36**	0.17
∑n-3 PUFA	**0.73**	0.12	**0.79**	0.14	**1.05**	0.01	**0.10**	0.01
∑n-6 PUFA	**0.67**	0.12	**0.71**	0.17	**48.98**	0.15	**9.21**	0.17
n-6/n-3	**0.92**	0.10	**0.90**	0.11	**46.64**	0.58	**88.98**	7.58

^1 ^Solid mush with just maize meal and water for analysis

^2 ^Palm oil-derived shortening

^3 ^Total digestible carbohydrates

### Statistical analysis

Statistical analyses were conducted via SSPS Statistics 17.0 for Microsoft^® ^Windows (SPSS Inc., Chicago, USA). The t-test was used to compare socio-economic data and dietary intake between blood donors and non-blood donors. Differences were considered significant at *P *< 0.05.

## Results

### Composition of diet based on 24-h recall

Based on the 24-h recall, all Maasai (n = 26) consumed sweetened milk tea in the morning. *Porridge *(a liquid mixture of water, maize meal [~3.5%], sugar [~5.5%] and cow's milk [~20%]) was consumed by a quarter of the Maasai, mainly women, around mid-morning. Lunch consisted of boiled cow's milk (n = 16) and *ugali *(n = 22). In addition, one third of the subjects consumed beans and one tenth had vegetables (cabbage or kale). Moreover, nearly half of the Maasai in the study drank sweetened milk tea in the afternoon. The evening meal was similar to lunch; the difference being that a greater variety of vegetables such as cabbage, maize, kale and potatoes were consumed.

In total, the Maasai consumed 793 ± 449 g fresh (boiled) cow's milk per day and person, of which 67% as direct consumption, 27% via sweetened milk tea, and 6% in the *porridge *(Table 2). According to the subjects sweetened milk tea was prepared with two thirds water, one third milk (mainly cow's milk), approximately 5% sugar, and a varying quantity of tea leaves. Only three subjects consumed fermented cow's milk during the course of the study. Thus, the average daily intake of fermented milk contributed to less than 10% (67 g/d) of total milk consumption. Furthermore, 582 ± 366 g/d of *ugali *were consumed (Table 2). Approximately 13 g/d of cooking fat were ingested, either as home-made *ghee *(7.3 ± 8.3 g) or in the form of 5.7 ± 10 g *Kimbo*^® ^(Table 2). Maasai women, who are generally in charge of buying and preparing the food, reported purchasing approximately 500 g of *Kimbo*^® ^per week. Home-made *ghee *was additionally used as baby food, as medicine for the chest and for stomach problems. Other types of fats, such as suet were consumed to a lesser extent (Table 2). Only one male Maasai ate beef (~125 g) during the two-week study period.

**Table 2:** Intake of foods in g per day and person based on 24-h recall (n = 26)

Food (g)	mean	SD
Fresh (boiled) cow's milk	**793**	449
Directly consumed	**533**	417
In tea	**216**	107
In porridge^1^	**45**	77
Fermented cow's milk	**67**	217
Goat's milk	**3.2**	16
Ugali^2^	**582**	366
Maize meal	**171**	108
Sugar	**42**	18
Rice (boiled)^3^	**44**	128
Chapatti^4^	**3.8**	20
Bread	**2.9**	15
Beans (boiled)^5^	**95**	126
Potatoes	**26**	64
Vegetables^6^	**95**	112
Fruits	**10**	49
Meat	**4.8**	25
Suet	**0.5**	1.6
Ghee^7^	**7.3**	8.3
Kimbo^®8^	**5.7**	10

^1 ^Liquid mush made up of water, maize meal, milk (sugar and cooking fat may be added)

^2 ^Solid mush consisting of maize meal and water (salt and fat may be added)

^3 ^Corresponded to 19 g uncooked rice

^4 ^Wheat flour flat pancake

^5 ^Corresponded to 33 g raw beans

^6 ^Cabbage, maize, kale, tomatoes, onions

^7 ^Homemade clarified butter

^8 ^Palm oil-derived shortening

The subjects were also asked as to when they had last drunk fermented cow's milk, animal blood and eaten meat. Here, the last intake of meat dated back to 12.8 ± 21.7 d. About 50% of the Maasai quoted that mutton had been their last meat consumption, 27% of them had eaten goat meat, and 15% beef. The remaining 8% did not specify the type of meat they had consumed. The last intake of fermented cow's milk dated back to 15.1 ± 33.3 d. Subjects explained that the frequency of intake of fermented milk depended on the season. While it was consumed almost daily during the wet season (i.e., November to December and April to June [8]), fermented milk was rarely consumed during the dry season. The last blood consumption dated back to 4.7 ± 7.5 months. Cattle blood in the form of the traditional dish *monono *(i.e., blood fried and boiled together with meat) had been eaten by 73% of Maasai, whilst 16% had ingested pure blood or blood mixed with milk, and 11% had never consumed blood.

### Daily intake of energy and nutrients based on 24-h recall

Daily energy intake of the Maasai according to the 24-h recall constituted 7.6 ± 2.1 MJ per day and person (9.5 ± 2.3/7.0 ± 1.8 MJ/d, males/females; Table 3). The diet consisted of 56.2 ± 9.4% carbohydrates, 13.3 ± 2.0% protein, and 30.5 ± 7.9% fat. This corresponded to a daily intake of 242 ± 73 g carbohydrates, 58 ± 21 g protein, and 59 ± 26 g fat. Of the total ingested fat, 46 g/d was dairy fat. Daily consumption of fibre and cholesterol amounted to 25 ± 14 g and 96 ± 65 mg, respectively.

**Table 3:** Contribution of foods and nutrients to the daily energy intake of Maasai in the current study compared to Nestel [16]

	Present study (n = 26^1^)			**Study of Nestel 1989 **[16] (n = 127^2^)		
	**Mean**	**SD**	**m/f**	**Mean**	**SD**	
		
**Energy intake (MJ)**	**7.6**	2.1	9.5/7.0	**5.1**	2.1	

	en%		g	en%		g^3^
		
**Contribution of nutrients**	**Mean**	**SD**	**m/f**	**Mean**	**SD**	

Carbohydrates	**56.2**	9.4	303/223	**48.4**	18.4	144.4
Protein	**13.3**	2.0	73/54	**14.8**	7.3	44.1
Fat	**30.5**	7.9	74/55	**35.8**	14.1	47.2
SFA (% of ∑ FAME)	**63.8**	6.5	47/35	**68.1**^4^	0.5	32
MUFA (% of ∑ FAME)	**27.1**	1.7	20/15	**28.0**^4^	0.3	13
PUFA (% of ∑ FAME)	**9.2**	0.6	7/5	**3.5**^4^	0.2	2
Cholesterol (mg)	**-**	**-**	120/89 mg	**-**	**-**	219 mg^4^
Alcohol	**0**	0	0	**0.9**	4.8	1.6
	en%		g	en%		g^3^
		
**Contribution of foods **	**Mean**	**SD**		**Mean**	**SD**	

*Dairy products*	***41.1***	*20.7*		***42.4***	*29.4*	
Milk^5^	37.5	20.9	863	41.2	30.3	690
Ghee	3.6	3.8	7.3	1.2	3.8	2

*Meat & suet*	***0.6***	*1.9*	*5.3*	***9.2***	*12.4*	*35*

*Plant-derived foods *	***58.3***	*20.7*		***48.4***	*12.1*	
Maize	33.0	17.4	171	22.2	23.6	79
Sugar	10.1	5.1	42	15.0	14.1	45
Other	15.2	- -		11.2	6.1	

f, females m, males

^1 ^20 female and 6 male subjects, Ø 33 years

^2 ^Kenyan Maasai (127 women, Ø 34 years; in total 273 food records)

^3 ^Calculated from the percentages

^4 ^Values from [7]

^5 ^All types of milk of which 793 g (34.5 en%) was fresh (boiled) cow's milk, 67 g (2.9 en%) fermented cow's milk and 3.2 g (0.1 en%) goat's milk

Cow's milk (fresh or boiled) and *ugali*, the two staples of the Maasai diet, contributed 34.5 ± 16.2% and 33.0 ± 17.4% to the total dietary energy intake (en%), respectively (Table 3). Sugar was mainly ingested via sweetened milk tea supplying 10.1 ± 5.1 en%, whereas beans and potatoes made up 7.0 ± 7.7 en%. Overall, the major proportion of the ingested food was plant-derived (58.3 ± 20.7 en%; Table 3).

Dietary fat was composed of 63.8% SFA, 27.1% MUFA, and 9.2% PUFA (Table 3). Palmitic acid and oleic acid were the predominant dietary fatty acids (32.1% and 21.5% of FAME; Table 4). In addition, subjects ingested 684 mg/d vaccenic acid (VA; C18:1 *t*11) and 474 mg/d of total CLA. However, only 84.0 mg/d of n-3 LC-PUFA (EPA, DPA and DHA) were consumed.

**Table 4:** Fatty acids in Maasai diet and red blood cells

	Maasai (present study)			
	**Diet** **(n = 26)**		**Red blood cells** **(n = 18)**	
**Fatty acids** **(% of total FAME)**	**mean**	**SD**	**mean**	**SD**

**C12:0**	**2.30**	0.65	**0.02**	0.01
**C14:0**	**8.66**	2.36	**0.42**	0.11
**C16:0**	**32.09**	2.66	**28.22**	1.66
**C16:1**	**1.49**	0.38	**0.50**	0.09
**C18:0**	**8.61**	1.56	**11.78**	1.53
**C18:1*cis*9**	**21.55**	4.18	**17.06**	1.39
**∑C18:1*trans***	**1.96**	0.55	**0.68**	0.14
**C18:1*t*11**	**1.20**	0.35	**0.29**	0.08
**C18:2n-6**	**7.15**	4.01	**12.93**	1.72
**∑CLA**	**0.83**	0.24	**0.54**	0.14
***c*9,*t*11 CLA**	**0.67**	0.20	**0.41**	0.12
**C18:3n-6**	**0.01**	0.00	**0.07**	0.02
**C18:3n-3**	**0.79**	0.25	**0.16**	0.04
**C20:0**	**0.35**	0.02	**0.18**	0.07
**C20:4n-6**	**0.04**	0.02	**13.66**	1.40
**C20:5n-3**	**0.05**	0.01	**0.84**	0.27
**C22:0**	**0.16**	0.03	**0.17**	0.07
**C22:5n-3**	**0.09**	0.03	**2.84**	0.38
**C22:6n-3**	**0.01**	0.00	**2.23**	0.64
**AA/EPA**	**0.84**	0.09	**16.3**	--

**∑SFA**	**63.79**	6.29	**44.07**	1.18
**∑MUFA**	**27.05**	2.97	**19.85**	1.37
**∑PUFA**	**9.16**	3.87	**36.08**	1.52
**∑n-3 PUFA**	**0.99**	0.23	**6.13**	0.86
**∑n-6 PUFA**	**7.24**	3.99	**29.29**	1.93
**n-6/n-3**	**7.38**	4.06	**4.91**	1.05

AA, arachidonic acid; EPA, eicosapentaenoic acid

### Distribution of fatty acids in red blood cells and concentrations of blood lipids

Red blood cells comprised 44.1% SFA, 19.9% MUFA, and 36.1% PUFA (Table 4). Palmitic acid was the most abundant fatty acid (28.2%). Further, proportions of the milk specific VA and CLA were found at 0.29% and 0.54%, respectively and the total n-3 PUFA fraction was 6.13%, consisting of 0.16% ALA, 0.84% EPA, 2.84% DPA, and 2.23% DHA (Table 4).

Mean plasma concentrations of TAG, total cholesterol, and LDL were similar for both genders and below the cut-off points set by the NCEP-ATP III ([15], Table 5). The mean HDL concentration for males was below the cut-off point at 1 mmol/L.

**Table 5:** Mean age, iron status and plasma blood lipids of the sub-group of blood donors

Parameter	Mean	SD	Mean	SD	**cut-off points**^**1**^
	male (n = 4)		female (n = 14)		
Age (years)^2^	38.5	23.2	31.4	9.5	-

Ferritin (μg/L)	70	47	20	17	> 15
Saturation of transferrin (%)	31	10	14	8	> 16
TAG (mmol/L)	1.2	0.5	0.9	0.4	< 1.7
total cholesterol (mmol/L)	4.3	0.5	4.3	1.1	< 5.2
LDL (mmol/L)	2.8	0.6	2.8	0.9	< 3.4
HDL (mmol/L)	0.9	0.1	1.1	0.2	> 1.0
LDL/HDL	3.3	1.1	2.7	0.8	

^1 ^For normal blood lipids according to NCEP-ATP III [15] and parameter of iron status below which iron stores are considered to be depleted (ferritin) or a marked anaemia is indicated (saturation of transferrin) according to the WHO [23]

^2 ^Only blood donors

## Discussion

The total milk intake of 863 g/d (fresh/boiled and fermented cow's milk and goat's milk) according to the 24-h recall was similar to that of earlier studies [16,17]. Nestel documented the mean annual milk consumption of Kenyan Maasai women as ~690 g/d (Table 3) [16]. Homewood et al. [17] reported a dry season milk intake of 809 g/African Adult Male Equivalent (AAME)/d for Tanzanian Maasai (August 1981, dry season), which meant that women (0.86 × AAME) consumed 696 g milk per person and day. The contribution of milk and milk products to the total energy intake in the present study (41.1 en%) was also comparable to that of Nestel (42.4 en%; Table 3) [16]. Moreover, fermented cow's milk was rarely consumed throughout the present study period (end of the short dry season, 67 g/d) which was analogous to Nestel's and Geissler's data reporting that Maasai seldom consumed soured or fermented milk throughout the year [18]. Even if fermented milk was consumed almost daily during the wet season (4.5 months vs. 7.5 months of dry season), as it was stated by the subjects in the current study, the average annual consumption is unlikely to reach 2 to 3 L per day and person as claimed by Mathara et al. [4].

The consumption of 582 g/d of *ugali *corresponded to 171 g/d of maize meal (amounting to 33 en%). This value was between the maize meal intake of Kenyan Maasai women (26 en%, 79 g/d, Table 3) [16] and Tanzanian Maasai men and women (53 en%, 292 g per AAME, i.e. women consumed 251 g/d) [17,19].

Although meat and blood are traditional constituents of the Maasai diet, they cannot be considered staple foods. The assertion of the Maasai that their last intake of blood dated back about 5 months (median 3.4) is consistent with previous findings indicating that blood is rarely consumed [1,8,17,20,21]. Based on the 24-h recall, the intake of meat in the present study comprised less than 1% (4.8 g/d) of the total energy (Table 2 &3). In contrast, during the 1980s, meat and animal fat contributed 10 en% and 8 en% to the total dietary energy of mixed gender Tanzanian Maasai [19] and Kenyan Maasai women [8], respectively. However, the present study period of two weeks (April 2007) was too short to document actual meat consumption and to determine the contribution of the large quantities of meat consumed during ceremonies to the total dietary energy intake [8,22]. Talle [22] reports that nowadays, in contrast to previous custom, some Maasai prefer to buy their meat instead of slaughtering their own livestock. Their own livestock is maintained as an important source of cash income. Brown [5] stated that selling of 1 kg of beef (live weight; 1000 kcal) in order to buy 2 kg of maize meal (8000 kcal) resulted in an energy gain of 8 to 1. For the Maasai, this reality has led to a progressive decline in the intake of meat.

Concentration of plasma ferritin and saturation of transferrin (Table 5) in Maasai men was above the required WHO threshold of 15 μg/L and 16%, respectively [23]. In contrast, the half of the Maasai women (n = 7) had a decreased ferritin concentration (< 15 μg/L, i.e. depleted iron stores). In addition, the saturation of transferrin of these seven women was below the threshold of 16%, indicating a marked anaemia [23]. The last meat intake of these women dated back 14.6 ± 15.1 d, in contrast to the remaining women and the Maasai men, whose last meat intake had been only 4.4 ± 2.1 d and 4.5 ± 3.9 d ago, respectively. These figures indicate that at least half of the Maasai women substantiate the observations of Brown [5] and Talle [22] of a declined meat intake.

The composition of the Maasai diet in this study corresponded to the mean annual composition of diets of African agro-pastoral tribes (including Kenyan and Tanzanian Maasai) of the dry tropics [24]. On average, these pastoral tribes obtain ~40 en% from the intake of milk and milk products and more than 50 en% from plant-derived products. In contrast, the supply of milk and egg in the average Kenyan population between 2000 and 2002 represented only 7% (235 g/d) of the total dietary energy source [25].

Among Maasai women and men, the energy intake of 7.0 MJ/d and 9.5 MJ/d, respectively, was below the recommended intake levels set by the WHO/FAO (in a developing country: rural women of 50 to 60 kg: 9.6 MJ/d to 10.0 MJ/d; healthy retired men of 80 kg: 11.4 MJ/d; subsistence farmers of 80 kg: 13.4 MJ/d [26]). Although, compared with the values reported by Nestel [16] (Table 3), the energy intake of women in the present study was higher.

The recommended protein intake set by WHO/FAO for both genders denotes 0.75 g protein/kg body weight [26]. Amongst Maasai of both genders, the protein intake was sufficient assuming that the average weight of women and men was 60 kg and 80 kg, respectively (Table 3). Nestel [16] reported an energy intake of 65% and a protein intake of 119% of the RDI adjusted for weight. According to Galvin [24], African pastoralists had a lower than required energy intake whereas their protein intake exceeded the reference value due to the high proportion of animal-derived foods in their diet, making them appear lean and tall.

As recommended by the WHO [27], carbohydrates made up > 55 en%, protein 10 to 15 en%, and fat 30 en% (Table 3). The dietary fat should ideally consist of less than 30% SFA and 20 to 30% PUFA [27]. The ingested fat of the Maasai determined in the current study, however, consisted of 63.8% SFA, 27.1% MUFA, and 9.2% PUFA. These values are in accordance with those reported by Nestel and Geissler [7] which were similarly high in SFA and low in PUFA (SFA/MUFA/PUFA: 68.1/28.0/3.5%, Table 3). The high proportion of dietary SFA is attributed to the high milk fat intake (Table 2 &3). Conversely, the cholesterol intake of both men (120 mg/d) and women (89 mg/d) was far below the threshold level of 300 mg/d [27]. Nestel and Geissler reported a higher cholesterol intake of 219 mg/d for Maasai women [7]. Allowing for the fairly low total energy and cholesterol intake, the increased SFA fraction consisting of 63.8% of the ingested fat, almost twice as high as the recommended value, appears to be without pathophysiological relevance, as neither TAG nor LDL-cholesterol concentrations were elevated in the study group (Table 5).

During the 1960s and 1970s, low plasma cholesterol concentrations were observed among the Maasai, despite a supposedly high intake of milk (3 to 5 L/d) and cholesterol (500 to 2000 mg/d) [1,2,28-30]. However, since the dietary data were not systematically documented, it is likely that the figures have been slightly overstated [31].

The steady milk intake of the Maasai (~860 g/d) also meant a high intake of the characteristic milk fatty acids, such as VA and CLA (1.2% and 0.8% of FAME). These circumstances were reflected by an enriched concentration of these fatty acids in red blood cells of the Maasai (0.3% and 0.5%, Table 5). Similarly elevated VA and CLA concentrations were also found in the red blood cells of Bulgarian shepherds (0.5% and 0.6% of FAME; [32]), a group that characteristically consumes high quantities of home-made ewe's milk products. In contrast, it has been shown that subjects consuming a ruminant-fat free diet for eight weeks had four to seven times lower concentrations of VA and CLA in their red blood cells (0.08% and 0.09%; [12]).

Although SFA (63.8%) made up almost two thirds of the dietary fat of the Maasai red blood cells comprised only 44% SFA (Table 4). Generally, the fatty acid distribution in human cell membranes such as red blood cells is believed to represent a useful biomarker for ascertaining the fatty acid supply, especially that of PUFA. However, according to results reported in a few interventional and observational studies (e.g., [33,34]), red blood cells poorly reflect dietary SFA, since SFA are also synthesized endogenously or preferably β-oxidized [35,36]. It is considered that the limited incorporation of SFA into cell membranes is necessary for purposes of biofunctionality [37].

With regard to concentrations of PUFA in red blood cells, it was established that despite the low dietary PUFA intake (9.2% of total fat), red blood cells were relatively rich in PUFA (36.1% of FAME; Table 4). Yet, the main foods of the Maasai - milk and *ugali - *are very low in n-3 LC-PUFA (Table 1). Moreover, n-3 LC-PUFA-rich foods, such as sea fish, were not consumed by the Maasai, a factor supported by previous results from Nestel and Kuipers [8,9]. In fact, the especially low intake of EPA and DHA (29 and 6 mg/d, respectively) of the Maasai is similar to that of vegans or vegetarians (10 to 21 mg/d; [38]). The n-3 LC-PUFA (EPA, DPA and DHA) dietary intake of the Maasai was more than three times lower (84 mg/d) compared to a German EPIC sub-cohort taking in ~300 mg/d n-3 LC-PUFA [39]. Nevertheless, the EPA and DPA contents in the red blood cells of the Maasai were similar to that of the German EPIC sub-cohort (EPA: 0.84% vs. 0.73%, DPA: 2.84% vs. 2.25%; [40]). The endogenous conversion from ALA to EPA and DPA has been reported as being limited at only 0.1 - 6%, and the conversion from ALA to DHA is supposedly even more limited (≤ 0.05%, [41]). Thus, there are indications that a continuously low intake of n-3 LC-PUFA results in an up-regulation of the endogenous synthesis of n-3 LC-PUFA from ALA as a precursor [38,42].

The DHA proportion in the red blood cells of the Maasai was 50% of the value in the German sub-cohort (2.23% vs. 4.60%; [40]), although the Maasai's intake of DHA was less than 5% compared to the German sub-cohort (6.2 mg/d vs. 170 mg/d; [39]). As mentioned above, although the endogenous conversion of ALA to DHA is restricted (≤ 0.05%, [41]), it seems to be increased under conditions of low dietary DHA intake [38,42]. But, an effective increment of DHA in human tissues is only achieved by direct DHA consumption, rather than by conversion from ALA to DHA [43].

Finally, due to the competitive effects of n-6 and n-3 PUFA, a low intake of n-6 PUFA (mainly linoleic acid) as seen in the diet of the Maasai (i.e., 1.7 en%) could be of advantage for n-3 LC-PUFA metabolism. Recently, Blasbalg et al. [44] reported that during the last century, consumption of n-6 PUFA (linoleic acid) in the Western diet, increased from 2.8 en% to 7.2 en% explaining the decrease of n-3 LC-PUFA in human tissues.

## Conclusions

The community of Maasai participating in the study consumed a milk- and maize meal-based diet in which plant-derived food accounted for more than half of their dietary energy. Total dietary fat consisted of a high proportion of SFA whilst levels of PUFA, especially of n-3 LC-PUFA, were fairly low. Despite this low intake, proportions of n-3 LC-PUFA in the red blood cells of the subjects were comparable with those in a German cohort ingesting higher quantities of these fatty acids.

## Abbreviations

ALA, α-linolenic acid: C18:3n-3; CLA, conjugated linoleic acids; DHA, docosahexaenoic acid: C22:6n-3; DPA, docosapentaenoic acid: C22:5n-3; en%; % of total dietary energy; EPA, eicosapentaenoic acid: C20:5n-3; FAME, fatty acid methyl esters; HDL, high density lipoprotein; LDL, low density lipoprotein; MUFA, monounsaturated fatty acids; n-3 LC-PUFA, omega-3 long-chain polyunsaturated fatty acids (C > 18); PUFA, polyunsaturated fatty acids; SFA, saturated fatty acids; TAG, triacylglycerides; TC, total cholesterol; VA, vaccenic acid

## Competing interests

The authors declare that they have no competing interests.

## Authors' contributions

NK developed the research proposal, designed the dietary questionnaire, analysed and interpreted the food and blood samples, evaluated the questionnaires and data, and drafted the manuscript. KK determined the CLA isomer distribution in food and blood samples, supervised analyses and interpretation of fatty acids and helped to draft the manuscript. FMK and BNKM co-developed the research proposal and conducted the dietary interviews. GJ conceived of the study, and participated in its design and coordination and helped to draft the manuscript. All authors read and approved the final manuscript.
